# Systematic Evaluation of Structure–Property–Biocompatibility Relationships of Polyhydroxyalkanoate Copolymers for Advanced Veterinary Applications

**DOI:** 10.3390/molecules31132375

**Published:** 2026-07-06

**Authors:** Kaijun Huang, Yaru Cao, Shuai Zhang, Yiming Sun, Miao Long, Suncheng’ai Cao, Xinyan Yang, Jiayi Wang, Ziyin Wang, Zhengyan Zhu, Shubiao Wu, Jianli Wang, Wenjian Ma, Lin Jin

**Affiliations:** 1 Shanxi Key Laboratory for Modernization of TCVM, College of Veterinary Medicine, Shanxi Agricultural University, Taiyuan 030031, China; 202410101@stu.sxau.edu.cn (K.H.);; 2 State Key Laboratory of Food Science and Resource, School of Biotechnology, Jiangnan University, Wuxi 214122, China; 3 School of Environmental and Rural Science, University of New England, Armidale, NSW 2351, Australia; swu3@une.edu.au

**Keywords:** polyhydroxyalkanoates, PHB, PHBV, P34HB, tunable degradation, biocompatibility, veterinary biomaterials

## Abstract

Polyhydroxyalkanoates (PHAs) are a family of microbial polyesters distinguished by their excellent biocompatibility and tunable degradation profiles. However, systematic evaluation of the interaction effect between their chemical structure, material properties, and biological performance remains limited, particularly in veterinary medicine applications. In this study, we conducted comprehensive in vitro and in vivo evaluations of five commercially available PHA (co)polymers: poly(3-hydroxybutyrate) (PHB); poly(3-hydroxybutyrate-co-4-hydroxybutyrate) containing 5 mol% or 15 mol% 4-hydroxybutyrate monomer (P34HB 5%, P34HB 15%); and poly(3-hydroxybutyrate-co-3-hydroxyvalerate) containing 5 mol% or 15 mol% 3-hydroxyvalerate monomer (PHBV 5%, PHBV 15%). Material characterization confirmed that the introduction of proper comonomers effectively reduced crystallinity and accelerated the degradation process. Biological assays demonstrated quite excellent cytocompatibility and hemocompatibility among all tested PHA materials. Notably, P34HB 5% nanoparticles promoted human umbilical vein endothelial cell (HUVEC) migration. In a 12-week murine subcutaneous implantation model, the PHBV 15% group exhibited the thinnest fibrous capsule formation and the mildest inflammatory response. Furthermore, intramuscular injection of nanoparticles revealed favorable muscle tissue compatibility in all groups. These results provided a co-polymer ratio-based justification for PHA selection, thus providing support for the application of these biomaterials in veterinary drug delivery platforms, wound dressings, and biodegradable implants.

## 1. Introduction

Over the past few decades, veterinary medicine has witnessed growing interest in the development and application of polymeric biomaterials derived from both natural and synthetic sources. These materials fall into two broad categories: natural biopolymers, such as collagen, fibrin, silk, and bacterially produced polyhydroxyalkanoates (PHAs); and synthetic biopolymers, including polylactic acid (PLA) and polycaprolactone (PCL) [1]. Natural biopolymers have garnered particular attention for veterinary wound dressings, controlled drug delivery systems, and biodegradable implants, owing to their favorable biocompatibility, low toxicity, and comparatively low energy requirements during production. Among biodegradable polymers investigated for veterinary applications, PHAs constitute a particularly versatile subclass of microbial polyesters that can serve as implants, drug carriers, or biomaterials in tissue engineering and wound management [2]. Notably, many of these materials can be produced using waste streams from other industrial processes, reducing resource consumption while addressing the growing demand for sustainable and environmentally responsible healthcare solutions in veterinary practice. Consequently, such materials hold considerable promise for advancing green veterinary medicine and enhancing clinical outcomes [3].

Microbial polyesters are distinguished primarily by their excellent biocompatibility—negligible toxicity and predictable degradation under physiological conditions. As naturally synthesized biopolymers, PHAs are environmentally compatible throughout their entire production–use–disposal cycle, embodying the convergence of waste valorization and biomedical progress [4]. Several studies have shown that PHAs elicit milder immune responses than widely used polyester alternatives such as PLA, poly(lactide-co-glycolide) (PLGA), and PCL [5,6,7]. PHAs constitute a family of naturally occurring polyesters synthesized by microorganisms, with mechanical properties ranging from rigid thermoplastics to flexible elastomers—attributes readily tailored through substrate selection, bacterial strain, and fermentation optimization [8]. The PHA family encompasses first-generation poly(3-hydroxybutyrate) (PHB), second-generation poly(3-hydroxybutyrate-co-3-hydroxyvalerate) (PHBV), third-generation poly(3-hydroxybutyrate-co-3-hydroxyhexanoate) (PHBHHx), and fourth-generation poly(3-hydroxybutyrate-co-4-hydroxybutyrate) (P34HB), each generation offering distinct mechanical and degradation profiles suited to specific biomedical applications.

PHB is the most common form of PHAs—a homopolymer of 3-hydroxybutyrate (3HB) with high crystallinity, pronounced brittleness, and relatively slow degradation kinetics [9]. PHB has been produced from agro-industrial waste streams, including cacao shells, cheese whey, wine residues, wood hydrolysates, and beet molasses, highlighting its potential as a sustainable feedstock-based material [10]. PHB has been approved by the U.S. Food and Drug Administration (FDA) for clinical investigations. Notably, its primary degradation product, 3HB, is a naturally occurring constituent of mammalian blood, alleviating concerns regarding immunogenicity or metabolic toxicity [11].

PHBV is a copolymer of 3-hydroxybutyrate (3HB) and 3-hydroxyvalerate (3HV) synthesized by microbial fermentation. The incorporation of 3HV units effectively mitigates the inherent brittleness of PHB, imparting improved flexibility and mechanical strength [11]. Higher 3HV content reduces crystallinity, thereby enhancing ductility, tensile strength, and elongation at break [12]; however, the precise influence of the 3HV molar ratio on in vivo host responses remains to be fully elucidated. In addition to its established industrial scalability, PHBV exhibits favorable immunotolerance and marked chemical inertness. Nonetheless, its broader application is hampered by several persistent drawbacks, including pronounced hydrophobicity, residual brittleness, limited impact resistance, and suboptimal thermal stability.

P34HB is a copolymer of 3-hydroxybutyrate (3HB) and 4-hydroxybutyrate (4HB) that has emerged as a commercially available fourth-generation PHA [13]. By modulating the 4HB monomer content, both the mechanical and thermal properties of the material can be precisely tuned. Increasing 4HB content drives a transition from rigid, semicrystalline thermoplastics to soft, elastomeric materials [14]. Moreover, 4HB incorporation accelerates degradation kinetics and further improves biocompatibility [15].

Metabolic engineering of PHA biosynthetic pathways enables precise control over comonomer incorporation, thereby facilitating the on-demand synthesis of materials with tailored physicochemical and biological properties. In this context, the comonomer molar ratio (e.g., 5% versus 15%) serves as a key parameter for tuning material performance across a continuous spectrum extending from rigid thermoplastics to soft elastomers [16]. This inherent tunability markedly expands the potential utility of PHAs relative to conventional bioplastics.

Current research on PHAs and related materials has largely focused on two principal directions: mechanical property optimization, including toughness enhancement through copolymer modification and crystallinity control, and isolated biocompatibility assessments, such as cytotoxicity screening and evaluation of inflammatory responses following subcutaneous implantation. Owing to their favorable biocompatibility, controllable biodegradability, and tunable mechanical properties, PHAs have been widely investigated for diverse biomedical applications, encompassing bone, cartilage, nerve, vascular, and skin tissue engineering, in addition to their established roles in surgical sutures and drug delivery systems [17,18,19]. In contrast, veterinary-specific investigations remain comparatively sparse, with existing studies predominantly confined to aquaculture and animal production settings. For example, Baruah et al. (2015) demonstrated that PHB could confer protection against *Vibrio campbellii* infection in *Artemia franciscana* via the induction of heat shock protein expression [20], whereas Wang et al. (2019) evaluated the effects of dietary PHB supplementation on growth performance in large yellow croaker and swine [21].

Relative to the breadth of human clinical research, systematic exploration of veterinary-oriented PHA materials is still in its early stages. Notably, comprehensive comparative studies that assess the integrated biological performance of PHB, PHBV, and P34HB across varying comonomer ratios under a standardized experimental framework remain lacking. Furthermore, several key biological phenomena remain inadequately characterized, including the pro-migratory effects of specific PHA (co)polymers such as P34HB on relevant cell types, systematic hemocompatibility profiles across the PHA family, and the in vivo fate of PHA nanoparticles within muscle tissue. These knowledge gaps currently constrain the rational design and precise application of PHAs in veterinary wound repair, implantable devices, and related therapeutic contexts.

Methodologically, existing studies on PHA biocompatibility have predominantly focused on single-material evaluations or human clinical applications. A systematic comparison of PHB, PHBV, and P34HB—encompassing different comonomer types and ratios—under identical experimental conditions remains limited. Moreover, these materials are typically investigated in either film or nanoparticle form, but rarely both, obscuring how morphology influences biological responses. Films represent the surface architecture of implantable devices, whereas nanoparticles serve as drug delivery vehicles; these distinct clinical configurations necessitate separate biocompatibility assessments.

Accordingly, this study was designed to evaluate five commercially available PHA materials—PHB, poly(3-hydroxybutyrate-co-4-hydroxybutyrate) containing 5 mol% 4-hydroxybutyrate (P34HB 5%), poly(3-hydroxybutyrate-co-4-hydroxybutyrate) containing 15 mol% 4-hydroxybutyrate (P34HB 15%), poly(3-hydroxybutyrate-co-3-hydroxyvalerate) containing 5 mol% 3-hydroxyvalerate (PHBV 5%), and poly(3-hydroxybutyrate-co-3-hydroxyvalerate) containing 15 mol% 3-hydroxyvalerate (PHBV 15%)—encompassing in vitro cytocompatibility, hemocompatibility, and in vivo biocompatibility. The resultant data are expected to provide an empirical basis for selecting optimal PHA (co)polymers in veterinary applications, such as short-term drug delivery systems and tissue implants, while concurrently offering a reference framework for mitigating environmental burdens associated with medical plastics.

## 2. Results and Discussion

### 2.1. Material Characterization

#### 2.1.1. Scanning Electron Microscopy (SEM)

**Figure 1:**
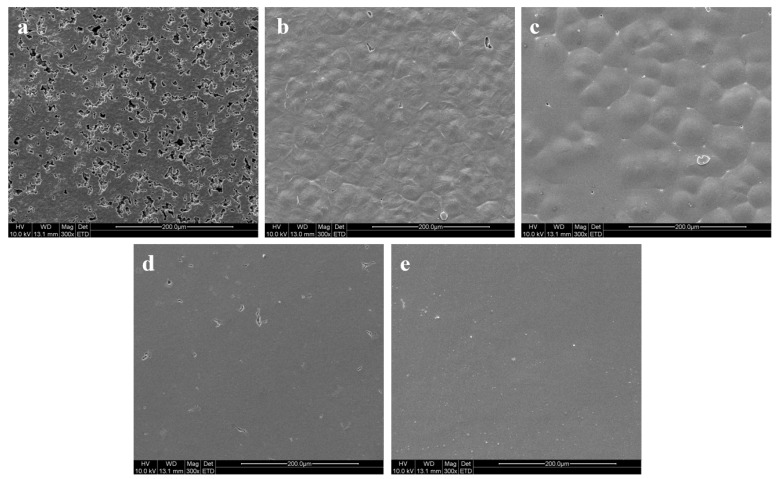
Surface morphology of PHB and its copolymers observed by SEM. (**a**) Poly(3-hydroxybutyrate) (PHB); (**b**) poly(3-hydroxybutyrate-co-4-hydroxybutyrate) containing 5 mol% 4-hydroxybutyrate (P34HB 5%); (**c**) poly(3-hydroxybutyrate-co-4-hydroxybutyrate) containing 15 mol% 4-hydroxybutyrate (P34HB 15%); (**d**) poly(3-hydroxybutyrate-co-3-hydroxyvalerate) containing 5 mol% 3-hydroxyvalerate (PHBV 5%); and (**e**) poly(3-hydroxybutyrate-co-3-hydroxyvalerate) containing 15 mol% 3-hydroxyvalerate (PHBV 15%). Magnification: 300×. Scale bar: 200 μm.

SEM imaging revealed marked differences in surface topography among the various PHA compositions (Figure 1). The PHB film exhibited a distinctly porous and rough architecture with heterogeneous pore distribution (Figure 1a), a morphology attributable to its high crystallinity and phase separation during solvent evaporation. In contrast, PHBV 5% and PHBV 15% films displayed relatively smooth and compact surfaces featuring only occasional micropores (Figure 1d,e), suggesting that the incorporation of 3HV comonomer effectively suppressed surface pore formation through a reduction in crystallinity. The P34HB films exhibited a distinct morphological progression: the 5% formulation displayed fine surface texturing (Figure 1b), whereas the 15% formulation developed regular cobblestone-like protrusions (Figure 1c). This topographical transition likely reflects the increased chain flexibility and altered self-assembly behavior conferred by a higher 4HB content.

Surface topography is known to critically influence cellular adhesion behavior. Prior studies have indicated that moderately rough, porous surfaces (exemplified by PHB) facilitate cell anchoring and spreading, whereas excessively smooth surfaces (as observed for PHBV) may compromise adhesion efficiency [22].

#### 2.1.2. Fourier Transform Infrared (FTIR) Spectroscopy

FTIR spectroscopy verified the characteristic absorption bands expected for PHAs, including the ester carbonyl (C=O) stretching band at 1718 cm^−1^ and C–O–C stretching modes of the ester linkage [23]. Relative to PHB, both PHBV and P34HB displayed an additional absorption feature at 2873–2874 cm^−1^, attributable to the ethyl side chains of 3HV or the methylene sequences of 4HB. Notably, increasing the comonomer content from 5% to 15% altered the relative intensities of crystallinity-sensitive bands at 1228 cm^−1^ (crystalline conformation) and 1182 cm^−1^ (amorphous conformation), accompanied by distinct peak broadening in P34HB 15% and PHBV 15%, indicating that comonomer incorporation reduced crystallinity and modified supramolecular packing. Full FTIR spectra and detailed peak assignments are provided in the Supplementary Materials (Figure S1, Table S1).

#### 2.1.3. Differential Scanning Calorimetry (DSC)

**Figure 2:**
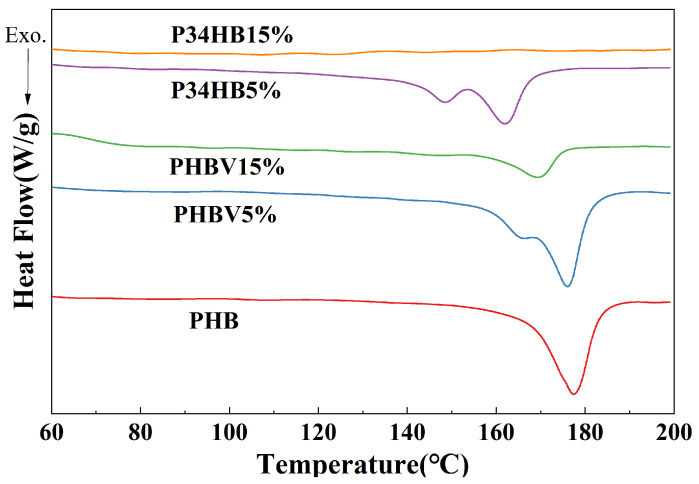
DSC second heating curves of PHA materials with different comonomer compositions.

DSC analysis revealed that comonomer incorporation substantially altered the thermal properties of the PHA materials (Figure 2; Table 1). To eliminate thermal history effects, data acquired during the second heating cycle were used for comparative analysis. Pure PHB exhibited a melting temperature (Tm) of 179.47 °C and a crystallinity of 67.49%. For P34HB, increasing the 4HB content from 5 mol% to 15 mol% progressively reduced Tm to 156.92 °C and 110.18 °C, while crystallinity decreased from 37.34% to 25.29%; notably, P34HB 15 mol% displayed a cold crystallization exotherm. Similarly, PHBV 15 mol% exhibited a Tm of 163.47 °C and a crystallinity of 17.90%. Collectively, these results demonstrate that both 4HB and 3HV comonomers disrupt the molecular chain regularity of PHB, thereby suppressing crystallinity in a concentration-dependent manner [24].

The observed reduction in crystallinity is consistent with the accelerated degradation kinetics and enhanced ductility documented in this study and aligns with the attenuated foreign body responses observed in the subsequent in vivo experiments. These findings underscore the feasibility of tailoring PHA materials for distinct clinical applications through rational modulation of the comonomer ratio [25]. While WAXD analysis would provide additional crystallographic detail, the combined DSC and FTIR data presented here consistently support the observed crystallinity-dependent trends in degradation and tissue response. WAXD characterization will be included in future studies to further clarify crystal structure effects on biological performance. Thermal parameters for all five formulations, including melting temperature, crystallinity, and cold crystallization data where applicable, are compiled in Table 1.

#### 2.1.4. Dynamic Light Scattering (DLS)

**Figure 3:**
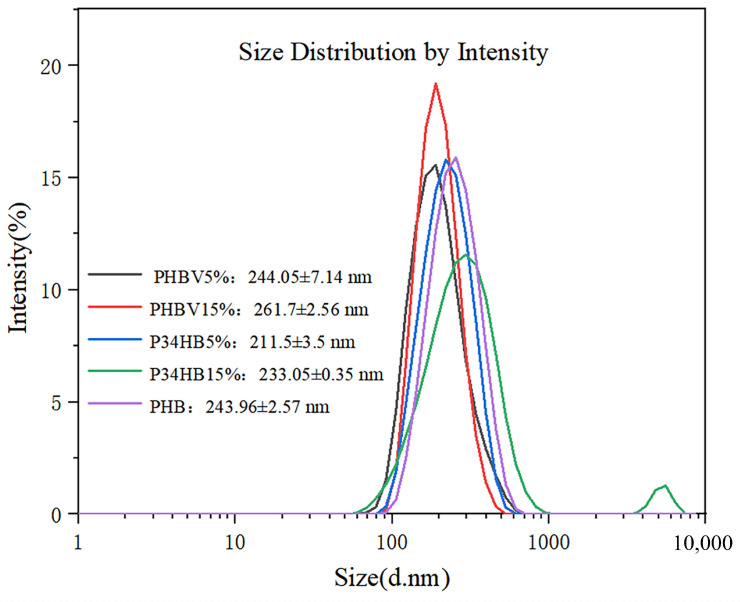
Size distribution of PHA nanoparticles with different compositions measured by DLS. Size (d.nm) represents the mean hydrodynamic diameter in nanometers.

Dynamic light scattering analysis revealed substantial differences in hydrodynamic diameter among the various PHA nanoparticle formulations (Figure 3; Table 2). PHB nanoparticles exhibited a mean diameter of 244.0 nm and a polydispersity index (PDI) of 0.228. The P34HB 5% formulation displayed the smallest particle size (211.5 nm) and the narrowest size distribution (PDI 0.207), reflecting favorable monodispersity. In contrast, PHBV 15% yielded the largest mean diameter (289.8 nm) and a comparatively broad distribution (PDI 0.336). Complete size and PDI data for all five formulations are summarized in Table 2.

Previous studies have demonstrated that nanoparticle size critically governs cellular uptake efficiency and in vivo biodistribution. Particles in the 200–300 nm range are readily recognized and internalized by macrophages, making them particularly suitable for vaccine delivery and targeted drug administration [26]. All formulations evaluated in this study exhibited PDI values below 0.4, confirming satisfactory colloidal stability and dispersion uniformity.

These results indicate that modulation of the comonomer ratio enables precise tailoring of nanoparticle dimensions to accommodate the specific demands of distinct administration routes. For example, intravenous injection requires smaller particles to minimize the risk of embolization, whereas intramuscular administration may benefit from larger particles that prolong retention at the injection site.

### 2.2. In Vitro Biological Evaluation

#### 2.2.1. Cell Viability Assessment (CCK-8 Assay)

**Figure 4:**
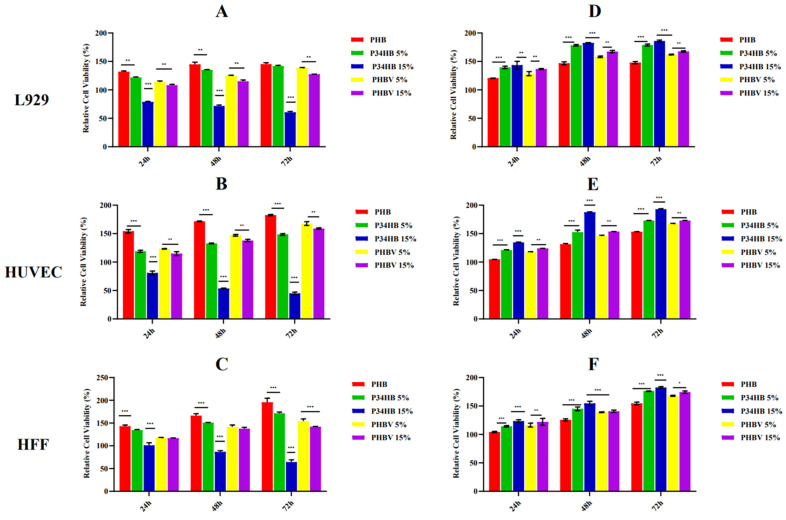
Cell viability of L929 murine fibroblasts (L929), human umbilical vein endothelial cells (HUVECs), and human foreskin fibroblasts (HFF) cultured with PHA materials. (**A**–**C**) Membrane samples; (**D**–**F**) Nanoparticle samples. Cell viability was determined by CCK-8 assay at 24, 48, and 72 h. Data are presented as Mean ± SD (*n* = 3), (* *p* < 0.05, ** *p* < 0.01, *** *p* < 0.001).

CCK-8 assays confirmed that PHB and its copolymers exhibited favorable cytocompatibility across all three cell lines evaluated—namely, L929 murine fibroblasts (L929), human umbilical vein endothelial cells (HUVECs), and human foreskin fibroblasts (HFF) (Figure 4). Among the film samples, PHB consistently yielded the highest metabolic activity at all time points, reaching 130–200% relative to the negative control (cells without material) within 24–72 h, and markedly outperformed the P34HB 15% group. The latter displayed comparatively lower activity in L929 cells, suggesting that elevated 4HB content may modify surface properties in a manner that attenuates initial cell adhesion. In contrast, nanoparticle formulations substantially enhanced cellular metabolic activity across all compositions tested, with P34HB 15% nanoparticles eliciting the most pronounced pro-proliferative effect on HUVECs.

Material morphology emerged as a critical determinant of cellular response. Film samples primarily governed cell adhesion and spreading through surface-mediated interactions, whereas nanoparticles exerted their effects via intracellular uptake and subsequent degradation. Prior work has demonstrated that PHA nanoparticles can be internalized by cells and undergo intracellular degradation in lysosomes [27]. 3-Hydroxybutyrate (3HB), a known degradation product of PHA materials, is a naturally occurring metabolic intermediate that serves as an energy substrate and supports cellular metabolic activity [28,29]. Cell line-dependent sensitivities to material composition were also apparent: L929 fibroblasts showed greater susceptibility to variations among P34HB film formulations, while HUVECs responded more favorably to the corresponding nanoparticles. This disparity likely reflects cell type-specific differences in nanoparticle internalization efficiency and intracellular trafficking [26].

Cell viability in all experimental groups remained above the 70% threshold prescribed by ISO 10993-5 [30], confirming that PHB, P34HB, and PHBV—whether evaluated as films or nanoparticles—fulfill the fundamental safety criteria for biomaterials. Viability values exceeding 100% in certain groups are indicative of the intrinsic pro-proliferative activity of PHA-based materials rather than any cytotoxic effect. These observations are fully consistent with the well-documented biocompatibility of the PHA family, whose hydrolytic degradation products are endogenous metabolites that do not provoke adverse tissue or cellular reactions [22].

#### 2.2.2. Cell Migration Assay

**Figure 5:**
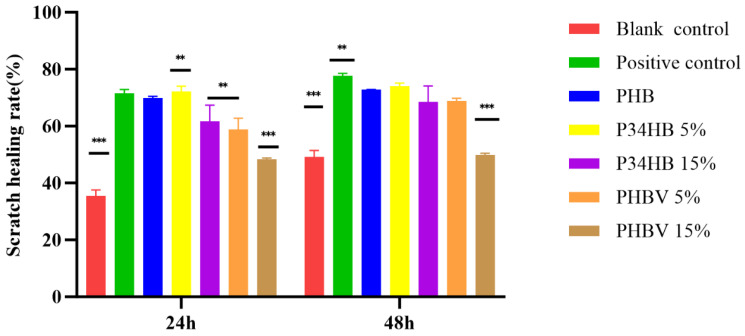
Quantitative analysis of HUVEC migration at 24 h and 48 h. Data are expressed as mean ± SEM (*n* = 3). ** *p* < 0.01, *** *p* < 0.001.

Scratch assay results demonstrated that, relative to the blank control, all nanoparticle formulations significantly accelerated HUVEC scratch closure at both 24 h and 48 h (Figure 5 and Figure S2), consistent with their non-cytotoxic profile established in the CCK-8 assay and indicative of enhanced endothelial cell migration. Notably, the wound healing rates achieved with P34HB 5% and PHB nanoparticles were comparable to those of the positive control, whereas the P34HB 15% and PHBV formulations exhibited markedly weaker pro-migratory effects. These findings are consistent with those reported by Yao et al., who documented that PHAs of varying monomer composition exert distinct influences on HUVEC behavior while maintaining favorable endothelial compatibility [31]. The superior performance of P34HB 5% observed in the present study may be attributable to the incorporation of 4HB units, which moderately reduces crystallinity and enhances chain flexibility, thereby establishing a microenvironment more permissive to cell migration.

#### 2.2.3. Hemolysis Assay

Hemolytic activity serves as a critical indicator of erythrocyte membrane integrity; accordingly, biomaterials intended for prolonged blood contact must exhibit excellent hemocompatibility. In this study, hemocompatibility was evaluated in accordance with the ASTM F756 [32] standard method [33]. Under this classification, materials are categorized based on their hemolysis rate as non-hemolytic (0–2%), slightly hemolytic (2–5%), or hemolytic (>5%).

**Figure 6:**
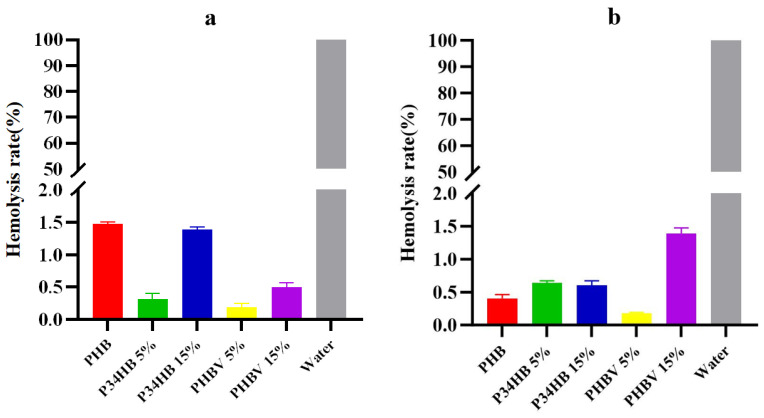
Hemolysis rates of PHA materials in different forms: (**a**) membrane samples; (**b**) nanoparticles.

Hemolysis assay results demonstrated that all tested materials—PHB, PHBV 5%, PHBV 15%, P34HB 5%, and P34HB 15%—whether evaluated as nanoparticles or films, exhibited hemolysis rates below 2% (Figure 6 and Figure S3), placing them firmly within the non-hemolytic category. These findings indicate negligible erythrocyte damage and support the potential utility of these materials in blood-contacting implantable devices.

Notably, the film formulations of PHB and P34HB 15% displayed marginally higher hemolysis rates than their nanoparticle counterparts. This modest elevation may be attributable to the larger continuous contact area and surface micro-roughness inherent to film architectures, which facilitate sustained interfacial interactions with erythrocytes. In contrast, nanoparticles remain well-dispersed in plasma and engage in comparatively milder contact with blood cells. Importantly, all film formulations remained below the 2% safety threshold, thereby satisfying the criteria for clinical applicability. These observations are consistent with previous reports demonstrating that PHA materials do not elicit clinically significant hemolytic reactions [34,35,36].

### 2.3. In Vivo Biological Evaluation

#### 2.3.1. Subcutaneous Implantation of Film Materials

This study systematically evaluated the in vivo biocompatibility of PHB and its copolymers (P34HB and PHBV) following subcutaneous implantation in C57BL/6 mice over a 12-week period. Hematological analysis revealed that white blood cell (WBC), red blood cell (RBC), hemoglobin (HGB), hematocrit (HCT), mean corpuscular hemoglobin (MCH), and platelet (PLT) counts in all experimental groups remained within normal physiological ranges throughout the observation period (Figure S4), with no evidence of material-associated hematotoxicity or exacerbated inflammatory responses. Blood biochemistry profiles further confirmed the absence of significant intergroup differences in key hepatic and renal function parameters, including aspartate aminotransferase (AST), alanine aminotransferase (ALT), alkaline phosphatase (ALP), and creatinine (Cr) (Figure S5), indicating that long-term implantation did not elicit systemic organ toxicity in the experimental animals.

**Figure 7:**
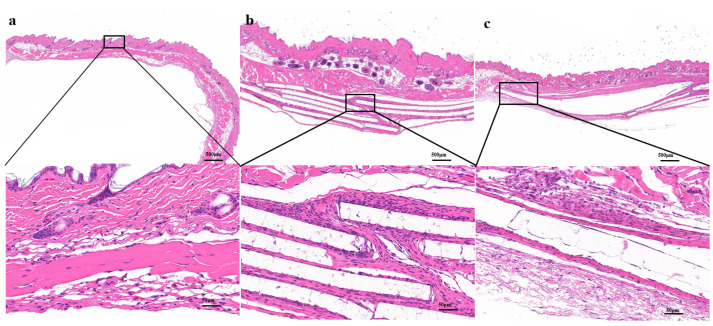
Representative H&E staining of skin tissue surrounding implanted PHA membranes at 12 weeks. **Upper panel**: low-magnification overview (scale bar = 500 μm); **lower panel**: high-magnification detail of boxed regions (scale bar = 50 μm). (**a**) PBS control, (**b**) PHB, (**c**) P34HB 5%.

**Figure 8:**
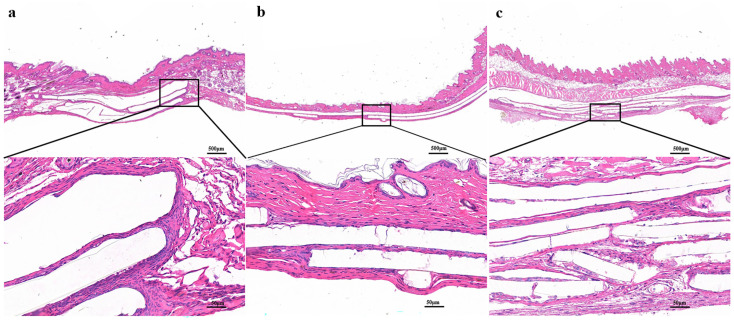
Representative H&E staining of skin tissue surrounding implanted PHA membranes at 12 weeks. **Upper panel**: low-magnification overview (scale bar = 500 μm); **lower panel**: high-magnification detail of boxed regions (scale bar = 50 μm). (**a**) P34HB 15%, (**b**) PHBV 5%, and (**c**) PHBV 15%.

Histological examination elucidated the regulatory effects of material composition on degradation behavior and tissue reactivity (Figure 7 and Figure 8). Hematoxylin and eosin (H&E) staining revealed that the pristine PHB implants maintained relatively intact structural integrity and were surrounded by a comparatively thick fibrous capsule with mild inflammatory cell infiltration. These observations are consistent with the high crystallinity and inherently slow degradation profile characteristic of PHB [37]. In contrast, the P34HB and PHBV copolymer groups exhibited accelerated degradation rates and attenuated tissue reactions. Notably, the PHBV 15% group demonstrated the most pronounced material resorption, the thinnest fibrous encapsulation, and the mildest inflammatory response among all formulations tested. These histological observations provide direct evidence of the composition-dependent in vivo structural stability of the implants over the 12-week implantation period. These phenomena are attributable to the disruption of molecular chain regularity and the consequent reduction in crystallinity conferred by comonomer incorporation, which collectively facilitate enzymatic hydrolysis and surface erosion [38]. Importantly, the skeletal muscle tissue underlying all implantation sites maintained normal histoarchitecture, with no evidence of myofiber degeneration, necrosis, or inflammatory infiltration (Figures S6 and S7), confirming that material implantation did not adversely affect deep muscular structures.

#### 2.3.2. Nanoparticle Intramuscular Injection Assessment

This study systematically evaluated the in vivo biocompatibility of PHB and its copolymers (P34HB and PHBV) following intramuscular injection of nanoparticles into the thigh muscles of C57BL/6 mice. Hematological analysis revealed that WBC, RBC, HGB, HCT, MCH, and PLT counts across all experimental groups (PBS control, PHB, P34HB 5%, P34HB 15%, PHBV 5%, and PHBV 15%) remained within normal physiological ranges (Figure S8), with no evidence of material-associated hematotoxicity or systemic inflammatory responses. Blood biochemistry profiles further confirmed the absence of significant intergroup differences in key hepatic and renal function parameters, including AST, ALT, ALP, creatinine, and urea (Figure S9), indicating that nanoparticle administration did not induce toxicological injury to major metabolic organs.

**Figure 9:**
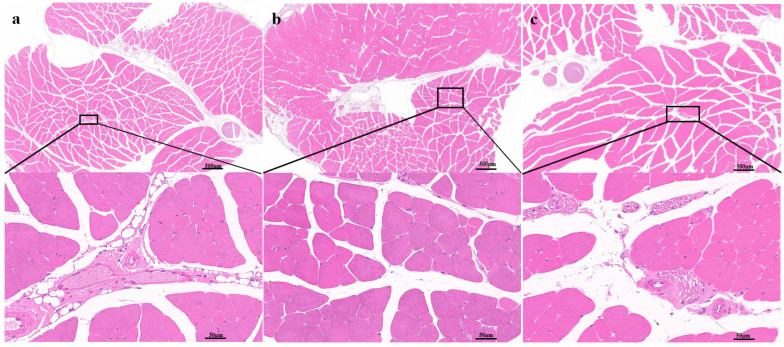
Representative H&E staining of skeletal muscle tissue at the injection site. **Upper panel**: low-magnification overview (scale bar = 500 μm); **lower panel**: high-magnification detail of boxed regions (scale bar = 50 μm). (**a**) PBS control, (**b**) PHB, (**c**) P34HB 5%.

**Figure 10:**
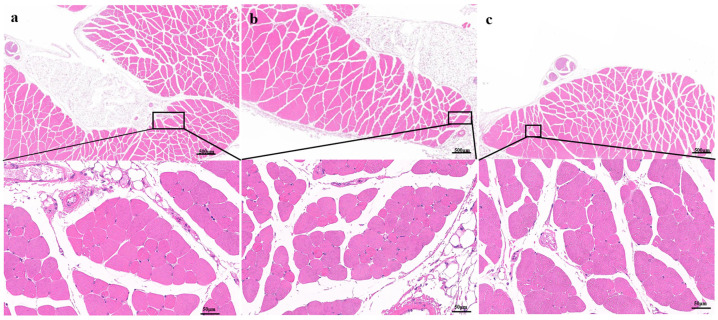
Representative H&E staining of skeletal muscle tissue at the injection site. **Upper panel**: low-magnification overview (scale bar = 500 μm); **lower panel**: high-magnification detail of boxed regions (scale bar = 50 μm). (**a**) P34HB 15%, (**b**) PHBV 5%, and (**c**) PHBV 15%.

Histological examination of muscle tissue (Figure 9 and Figure 10) revealed that the PBS control group (panel a) exhibited regularly arranged polygonal myofibers with normal intermuscular connective tissue and no evidence of inflammation or necrosis. The PHB group (panel b) displayed slight interstitial widening between myofibers and focal inflammatory cell infiltration at the injection site, suggesting that pristine PHB nanoparticles may elicit a mild foreign body response; however, no overt myofiber degeneration or necrosis was observed. Both the P34HB 5% (panel c) and P34HB 15% (panel d) groups maintained muscle architecture comparable to that of the control, with intact myofiber morphology and minimal inflammatory infiltration, indicating that the incorporation of 4HB units improved material biocompatibility. Similarly, the PHBV 5% (panel e) and PHBV 15% (panel f) groups exhibited unremarkable muscle architecture without discernible pathological alterations. These observations are consistent with the findings of Juni and Nakano, who reported transient acute inflammatory responses following intramuscular injection of PHB microspheres into rat thigh muscle, with inflammation resolving within seven days post-injection [39].

This study provides a systematic evaluation of PHB and its copolymers, P34HB and PHBV, as candidate biomaterials for veterinary applications. By evaluating all five formulations under standardized conditions, this study enables direct comparison of structure–property–biocompatibility relationships rarely addressed in prior single-material reports. The dual-morphology approach further reveals distinct cellular response patterns that would remain undetected if either configuration were omitted. In vitro characterization confirmed that modulation of the comonomer ratio enables precise control over thermal properties, degradation kinetics, and nanoparticle size, thereby accommodating the specific requirements of distinct administration routes. In vivo implantation studies further demonstrated that comonomer incorporation accelerates material degradation and attenuates the host tissue response. Notably, PHBV 15% elicited the thinnest fibrous capsule formation and the mildest inflammatory reaction following subcutaneous implantation, whereas intramuscular injection of nanoparticles revealed favorable tissue compatibility across all formulations, with only pristine PHB provoking a mild foreign body response.

These findings reveal divergent structure–function relationships between the P34HB and PHBV copolymer series: 4HB-containing copolymers appear to favor cellular migration and endothelial interactions, whereas 3HV incorporation may be more effective in mitigating the long-term foreign body response in subcutaneous tissue. Precise control over degradation behavior and tissue reactivity can be achieved through modulation of the comonomer ratio, thereby enabling rational material selection tailored to specific veterinary clinical scenarios. P34HB 15%, which exhibited the most rapid degradation kinetics and the mildest inflammatory profile, is well suited for short-term resorbable applications, such as drug carriers and wound dressings requiring a degradation window of approximately 2–3 weeks. P34HB 5% and PHBV 15%, both characterized by intermediate degradation rates and favorable biocompatibility, are appropriate for medium-term implants, including sutures and soft tissue patches with an anticipated resorption period of 4–8 weeks. PHBV 5%, owing to its slower degradation and prolonged maintenance of mechanical integrity, is indicated for medium-to-long-term supportive devices, such as orthopedic fixation hardware or vascular scaffolds that necessitate functional persistence over 3–6 months. Although PHB elicited pronounced fibrous encapsulation and a discernible foreign body response upon subcutaneous implantation, intramuscular injection of PHB nanoparticles provoked only mild, transient inflammation. Consequently, PHB may be considered for short-term intramuscular drug depot formulations but is not recommended for long-term soft tissue implantation.

Poly(4-hydroxybutyrate) (P4HB), a member of the PHA family, has received FDA approval for use in absorbable sutures, as exemplified by the commercial product TephaFLEX^®^. Its favorable tensile strength and extended degradation profile of approximately 12–18 months render it well suited for soft tissue repair procedures, thereby eliminating the stress and infection risks associated with secondary suture removal [40]. In orthopedic applications, the piezoelectric properties of PHBV have been shown to promote osteogenesis, making this copolymer a viable candidate for internal fracture fixation and joint repair in small animals. Notably, the degradation products of PHBV are less acidic than those of PLA, thereby attenuating the risk of localized inflammatory complications [41]. The pronounced flexibility of P34HB, meanwhile, renders it an attractive option for cardiovascular stent coatings [13]. Beyond these macroscopic applications, PHA nanoparticles hold considerable promise as controlled-release carriers for antimicrobial agents or vaccines. Such formulations could enable long-acting therapeutic interventions in companion animals via intramuscular administration, reducing dosing frequency and improving compliance. In livestock production settings, similar nanoparticle-based strategies may facilitate parasite control in ruminants, thereby enhancing overall production efficiency [42].

Compared with petroleum-derived plastics, PHAs are produced through microbial fermentation and are therefore both renewable and fully biodegradable—attributes that align closely with the growing emphasis on environmental sustainability in veterinary practice. This study provides an experimental foundation for the rational deployment of PHB-based materials in veterinary clinical settings, demonstrating that copolymer modification enables the tailoring of material properties to meet the demands of distinct species and therapeutic scenarios. Enzymatic degradation kinetics were not assessed in this study; future work should systematically compare the degradation rates of PHA films and nanoparticles under physiologically relevant enzymatic conditions. Future work should aim to elucidate the long-term degradation behavior and host immune responses of these materials in relevant animal models, while concurrently addressing challenges associated with scalable manufacturing processes, thereby accelerating the clinical translation of this class of sustainable biomaterials in veterinary medicine.

## 3. Materials and Methods

### 3.1. Experimental Materials

#### 3.1.1. Cells

L929 murine fibroblasts, human umbilical vein endothelial cells (HUVECs), and human foreskin fibroblasts (HFFs) were obtained from our laboratory collection and maintained in-house under standard culture conditions. HUVECs were cultured in Dulbecco’s Modified Eagle Medium (DMEM; Gibco, Waltham, MA, USA) supplemented with 10% fetal bovine serum (FBS; ExCell Bio, Suzhou, China) and 1% penicillin/streptomycin solution (P/S; Gibco). L929 cells were maintained in RPMI-1640 medium (Gibco) supplemented with 10% FBS, 1% P/S, 1% non-essential amino acids (NEAA; Gibco), 0.1% β-mercaptoethanol (Gibco), and 0.1% sodium pyruvate (Gibco). HFFs were cultured in DMEM (Gibco) supplemented with 10% FBS, 1% P/S, and 10 mM HEPES buffer. All cells were incubated at 37 °C in a humidified atmosphere containing 5% CO_2_.

#### 3.1.2. Animals

Female C57BL/6J mice (6 weeks of age) were obtained from the Laboratory Animal Center of Shanxi Provincial People’s Hospital (Taiyuan, Shanxi, China). Animals were group-housed under controlled environmental conditions at 22 ± 1 °C with a 12 h light/dark cycle, and were provided with sterilized water and standard rodent chow ad libitum. Animals were allowed a minimum acclimatization period of 7 days prior to any experimental procedures. All animal procedures were approved by the Animal Care and Use Committee of Shanxi Agricultural University (Approval No. SXAU-EAW-2025M.CR.005011472).

#### 3.1.3. PHA Raw Materials

The PHA materials used in this study included PHB, PHBV with 3-hydroxyvalerate contents of 5 mol% (PHBV 5%) and 15 mol% (PHBV 15%), and P34HB with 4-hydroxybutyrate contents of 5 mol% (P34HB 5%) and 15 mol% (P34HB 15%). All materials were supplied by PhaBuilder Biotechnology Co., Ltd. (Beijing, China). Prior to use, samples were stored in a desiccator at room temperature under light-protected conditions.

#### 3.1.4. Preparation of PHA Films

PHB, P34HB (5 mol% and 15 mol% 4HB), and PHBV (5 mol% and 15 mol% 3HV) were individually dissolved in chloroform to prepare 2% (*w*/*v*) solutions. The solutions were placed in amber glass bottles and heated at 80 °C for 3 h until complete dissolution. After cooling, 10 mL of each solution was slowly and uniformly poured into a 9 cm diameter glass Petri dish. The dishes were covered with microporous sealing film (0.5 mm pore size), sealed, and initially maintained at 60 °C for 1 h, followed by solvent evaporation at room temperature for 12 h. Films exhibiting uniform morphology and structural integrity were selected, immersed in 75% ethanol overnight, rinsed with phosphate-buffered saline (PBS), and sterilized by ultraviolet irradiation prior to use.

#### 3.1.5. Preparation of PHA Nanoparticles

Nanoparticles were prepared by an emulsion solvent evaporation method. Poly(vinyl alcohol) (PVA) was dissolved in deionized water to obtain a 10 g/L aqueous solution. Each of the five polymers was separately dissolved in chloroform with heating at 80 °C to serve as the oil phase. The oil phase was drawn into a syringe and added dropwise into the PVA solution at a 1:5 volume ratio under continuous sonication (38% of maximum power). After 10 min of sonication, additional PVA solution was introduced to reach the target volume, and the emulsion was subjected to further sonication using an ultrasonic cell disruptor for 20 min. The resulting emulsion was stirred at room temperature for 12 h to allow complete evaporation of chloroform. The nanoparticle suspension was initially centrifuged at 9000 r/min for 10 min to remove unencapsulated PVA and large aggregates. The supernatant was then centrifuged at 10,000 r/min for 20 min to collect the nanoparticles. The pellet was washed two to three times with PBS. The purified nanoparticles were lyophilized, sealed, and stored under desiccated conditions at 4 °C or −20 °C. Immediately prior to use, the nanoparticles were resuspended in PBS or culture medium to the desired concentration.

### 3.2. Material Characterization

#### 3.2.1. Scanning Electron Microscopy (SEM)

SEM imaging was performed using an SU8220 cold field emission scanning electron microscope (Hitachi High-Technologies Corporation, Tokyo, Japan) operated at an accelerating voltage of 15 keV. Prior to analysis, all samples were sputter-coated with a thin gold layer to enhance surface conductivity.

#### 3.2.2. Fourier Transform Infrared (FTIR) Spectroscopy

FTIR spectra were acquired using a Nexus series Fourier transform infrared spectrometer (Thermo Nicolet Corporation, Madison, WI, USA) operated with OMNIC software (v9.2), employing the KBr pellet method. Spectra were recorded over the range of 4000–600 cm^−1^ at a spectral resolution of 4 cm^−1^, with 32 scans accumulated per sample.

#### 3.2.3. Differential Scanning Calorimetry (DSC)

DSC analysis was performed using a TA Q200 differential scanning calorimeter (TA Instruments, New Castle, DE, USA) operated with TA Universal Analysis software (v4.5A). Approximately 5 mg of each sample was sealed in a standard aluminum pan. Under a nitrogen atmosphere, samples were heated from room temperature to 200 °C at a ramp rate of 10 °C/min, held isothermally for 5 min to erase thermal history, and then cooled to 50 °C at 10 °C/min. A second heating scan was subsequently conducted from room temperature to 200 °C at 10 °C/min. The melting temperature (Tm) and enthalpy of fusion (ΔHm) were determined from the second heating thermogram. Relative crystallinity (Xc) was calculated according to the following equation:

Xc (%) = (ΔHm/ΔHm^0^) × 100%

where ΔHm is the measured enthalpy of fusion of the sample, and ΔHm^0^ is the theoretical enthalpy of fusion for 100% crystalline PHB, taken as 146 J/g [43].

#### 3.2.4. Dynamic Light Scattering (DLS)

Prepared nanoparticles were dispersed in PBS to obtain a 1 mg/mL suspension, followed by sonication for 10 min at 300 W to disrupt aggregates. Large particulates were removed by filtration through a 0.22 μm hydrophilic membrane, and the nanoparticle concentration in the filtrate was verifiedusing a UV-2600 ultraviolet-visible spectrophotometer (Shimadzu, Kyoto, Japan) operated with UVProbe software (v2.70). The sample was transferred to a disposable cuvette and equilibrated at 25 °C. Particle size distribution was measured using a Zetasizer Nano ZS instrument (Malvern Panalytical, Malvern, UK) operated with Zetasizer Software (v7.13) in backscatter detection mode.

### 3.3. In Vitro Biological Evaluation

#### 3.3.1. Cell Viability Assessment (Cell Counting Kit-8 Assay)

The Cell Counting Kit-8 (CCK-8) assay was employed to evaluate the effects of PHB, PHBV 5%, PHBV 15%, P34HB 5%, and P34HB 15%—in both film and nanoparticle formulations—on cell proliferation. L929 murine fibroblasts, human umbilical vein endothelial cells (HUVECs), and human foreskin fibroblasts (HFFs) were used for this assessment. Film samples were punched into circular disks compatible with 96-well plates, sterilized by immersion in 75% ethanol followed by ultraviolet irradiation for 1 h, and rinsed twice with sterile PBS prior to use. Nanoparticle samples were dispersed in serum-free medium to prepare a 1 mg/mL stock suspension, sonicated to ensure homogeneity, filtered through a 0.22 μm membrane, and diluted to a final concentration of 50 μg/mL in complete culture medium immediately before application.

Cells were seeded into 96-well plates at a density of 5 × 10^3^ cells per well. After 24 h of culture to allow cell adhesion, the medium was replaced with nanoparticle-containing medium (50 μg/mL) for the nanoparticle groups; for the film groups, sterilized film disks were placed at the bottom of the wells prior to cell seeding. To minimize edge effects, the peripheral wells of each plate were filled with 100 μL of PBS. Each experimental group was assayed in triplicate, and blank controls (culture medium only) and negative controls (cells cultured without test materials) were included in each independent experiment. Following co-culture for 24, 48, and 72 h, 10 μL of CCK-8 reagent was added to each well, and the plates were incubated at 37 °C for 2 h in the dark. Absorbance was measured at 450 nm using a microplate reader. Cell viability was calculated using the following equation:

Cell viability (%) = [(ODexperimental − ODblank)/(ODnegative control − ODblank)] × 100%

#### 3.3.2. Cell Migration Assay (Scratch Assay)

HUVECs were seeded into 6-well plates at a density of 8 × 10^5^ cells per well and cultured in complete DMEM supplemented with 10% FBS until reaching >95% confluence. A linear scratch wound was created across the cell monolayer using a sterile 200 μL pipette tip. After gentle washing with PBS to remove detached cells and debris, the culture medium was replaced with serum-free DMEM containing 50 μg/mL nanoparticles. The nanoparticle formulations (PHB, PHBV 5%, PHBV 15%, P34HB 5%, and P34HB 15%; 100–200 nm) were pre-dispersed by sonication (300 W, 30 s) and filtered through a 0.22 μm hydrophilic membrane immediately before use. Negative control wells received serum-free DMEM only, whereas positive control wells were maintained in complete DMEM containing 20% FBS. Images of identical microscopic fields were acquired at 0, 24, and 48 h using an inverted microscope. Cell migration was quantified by measuring the residual wound width and calculating the percentage of wound closure relative to the initial wound area.

#### 3.3.3. Hemolysis Assay

The hemocompatibility of PHB, PHBV 5%, PHBV 15%, P34HB 5%, and P34HB 15%—in both film and nanoparticle formulations—was evaluated using a hemolysis assay. Film samples were punched into circular disks compatible with 96-well plates, sterilized by immersion in 75% ethanol for 24 h, rinsed with PBS, and subjected to overnight ultraviolet irradiation. Each sterilized film was subsequently placed in 500 μL of 0.9% physiological saline for storage prior to testing. Nanoparticle samples were dispersed in PBS to prepare a 1 mg/mL suspension, sonicated at 300 W for 10 min to disrupt aggregates, and filtered through a 0.22 μm hydrophilic membrane to remove large particulates.

Fresh rabbit blood was collected into 10% sodium citrate as an anticoagulant and diluted to a 5% (*v*/*v*) erythrocyte suspension using 0.9% physiological saline. Experimental groups included a negative control (physiological saline), a positive control (deionized water), nanoparticle groups (final concentration 0.1 mg/mL), and film groups, with each condition assayed in triplicate. A 500 μL aliquot of the erythrocyte suspension was gently mixed with an equal volume of sample or control solution in a 1.5 mL centrifuge tube. After incubation at 37 °C for 60 min, the mixtures were centrifuged at 12,000 r/min for 10 min. Following visual inspection of the supernatant, 500 μL of the supernatant was collected and its absorbance measured at 545 nm using aSpectraMax M2 microplate reader (Molecular Devices, San Jose, CA, USA) operated with SoftMax Pro software (v7.1). The hemolysis rate was calculated using the following equation:

Hemolysis rate (%) = [(ODsample − ODnegative control)/(ODpositive control − ODnegative control)] × 100%

### 3.4. In Vivo Biological Evaluation

#### 3.4.1. Subcutaneous Implantation of Film Materials

Six-week-old female C57BL/6J mice (*n* = 4 per group), weighing 16–20 g, were used for this study. Group sizes were determined based on prior experience with subcutaneous implantation studies, and no formal sample size estimation was performed. Six experimental groups were established: PHB, PHBV 5%, PHBV 15%, P34HB 5%, P34HB 15% film implantation groups, and a PBS sham-operated control group. Film samples were cut to appropriate dimensions, disinfected by immersion in 75% ethanol, rinsed two to threetimes with sterile PBS to remove residual ethanol, sterilized by ultraviolet irradiation for 30 min, and air-dried prior to implantation.

Surgical procedures were performed under aseptic conditions with inhalational isoflurane anesthesia (4% for induction, 2% for maintenance). An incision of approximately 0.5 cm was made in the dorsal subcutaneous fascia, and the sterilized film samples were implanted (the sham-operated group received only the incision without material placement). Wounds were closed with absorbable sutures, and the surgical site was disinfected locally for three consecutive days postoperatively. Wound healing progression and general health status were monitored regularly throughout the experimental period. At 12 weeks post-implantation, the animals were euthanized for sample collection. The implanted films along with the surrounding skin and muscle tissue were harvested, fixed in 4% paraformaldehyde, embedded in paraffin, sectioned, and stained with hematoxylin and eosin (H&E). Blood samples were collected via retro-orbital bleeding from each group for complete blood count analysis (using EDTA-K_2_ as an anticoagulant) and for the assessment of hepatic and renal function. Histological analysis was performed by a pathologist blinded to group allocation.

#### 3.4.2. Nanoparticle Intramuscular Injection Assessment

Six-week-old female C57BL/6J mice (*n* = 4 per group) were randomly assigned to a control group (PBS) and five experimental groups (PHB, P34HB 5%, P34HB 15%, PHBV 5%, and PHBV 15%). Nanoparticle suspensions (1 mg/mL) were prepared using the emulsion solvent evaporation method. Under isoflurane anesthesia, a single 50 μL injection of the suspension was administered into the bilateral quadriceps femoris muscles of each mouse. The observation period was set at 7 days. At the experimental endpoint, blood samples were collected via retro-orbital bleeding for complete blood count analysis (using EDTA-K_2_ as an anticoagulant) and for the assessment of hepatic and renal function. Animals were subsequently euthanized, and the quadriceps femoris muscles at the injection sites were carefully dissected, fixed in 4% paraformaldehyde, embedded in paraffin, sectioned, and stained with hematoxylin and eosin (H&E) for histological evaluation of local inflammatory responses and systemic toxicity. Histological analysis was performed by a pathologist blinded to group allocation.

### 3.5. Statistical Analysis

The experimental data were organized using Microsoft Excel and analyzed with GraphPad Prism 10.1.2 (GraphPad Software, La Jolla, CA, USA). Continuous data are expressed as mean ± standard error of the mean. Intergroup comparisons were performed using two-tailed Student’s *t*-tests or two-way analysis of variance (ANOVA) followed by appropriate multiple comparisons correction. A *p*-value < 0.05 was considered statistically significant (* *p* < 0.05, ** *p* < 0.01, *** *p* < 0.001), whereas * *p* ≥ 0.05 indicated no significant difference. Normality was assessed using the Shapiro–Wilk test. When normality assumptions were not met, appropriate non-parametric tests were applied.

## 4. Conclusions

This study provides a systematic evaluation of the structure–property–biocompatibility relationships of five PHA-based materials with tunable degradation characteristics. Through comprehensive in vitro and in vivo characterization, we demonstrate that the comonomer ratio serves as a critical parameter for precisely tailoring thermal properties, degradation kinetics, and host biological responses. All tested materials satisfied the biocompatibility criteria established by ISO 10993-5 (cytotoxicity) and ASTM F756 (hemocompatibility). Relative to the PHB homopolymer, the copolymer formulations (PHBV and P34HB) exhibited accelerated degradation kinetics and attenuated inflammatory reactions. Notably, PHBV 15% achieved optimal tissue integration, as evidenced by minimal fibrous capsule formation, whereas P34HB 5% markedly promoted endothelial cell migration, indicating superior potential for vascular-related applications.

On the basis of these findings, we propose a scientific framework to guide the rational selection of materials for specific veterinary clinical scenarios: P34HB 15% is indicated for short-term resorbable applications (approximate degradation window of 2–3 weeks), PHBV 15% and P34HB 5% are appropriate for medium-term implants (4–8 weeks), and PHBV 5% is suitable for long-term supportive devices (3–6 months). This work advances the rational design of sustainable biopolymers for veterinary medicine, contributing to environmentally responsible healthcare solutions while reducing the burden of medical plastic waste. Future studies should extend to large animal models and explore functionalization strategies to enable targeted therapeutic applications.

## Figures and Tables

**Table 1:** DSC data for PHA materials with varying comonomer contents (crystallization temperature upon cooling, Tc; melting temperature, Tm; enthalpy of fusion, ΔHm; Relative crystallinity, Xc. All data obtained from the second heating cycle).

Sample	Tc (°C)	Tm (°C)	ΔHm (J/g)	Xc (%)
PHB	84.18	179.47	98.53	67.49
P34HB 5%	79.55	156.92	54.52	37.34
P34HB 15%	117.85	110.18	36.93	25.29
PHBV 5%	78.59	175.35	51.88	35.53
PHBV 15%	118.11	163.47	26.13	17.9

**Table 2:** Hydrodynamic diameter and polydispersity index of PHA nanoparticles.

Sample	Z-Average (nm)	PDI
PHB	244.0 ± 2.1	0.228 ± 0.015
P34HB 5%	211.5 ± 2.9	0.207 ± 0.007
P34HB 15%	219.1 ± 19.8	0.345 ± 0.032
PHBV 5%	232.1 ± 17.4	0.298 ± 0.036
PHBV 15%	289.8 ± 43.1	0.336 ± 0.030

## Data Availability

The original contributions presented in this study are included in the article and Supplementary Materials. Further inquiries can be directed to the corresponding author.

## Supplementary Materials

The following supporting information can be downloaded at: https://www.mdpi.com/article/10.3390/molecules31132375/s1. Figure S1. FTIR spectra of PHB and its copolymers in the range of 3100–500 cm^−1^; Figure S2. Representative images of HUVEC scratch wound healing at 0, 24, and 48 h. Scale bar = 200 μm. Poly(3-hydroxybutyrate) (PHB); poly(3-hydroxybutyrate-co-4-hydroxybutyrate) containing 5 mol% 4-hydroxybutyrate (P34HB 5%); poly(3-hydroxybutyrate-co-4-hydroxybutyrate) containing 15 mol% 4-hydroxybutyrate (P34HB 15%); poly(3-hydroxybutyrate-co-3-hydroxyvalerate) containing 5 mol% 3-hydroxyvalerate (PHBV 5%); and poly(3-hydroxybutyrate-co-3-hydroxyvalerate) containing 15 mol% 3-hydroxyvalerate (PHBV 15%).; Figure S3. Nanoparticle morphology after incubation. Representative images of hemolysis assay after incubation with red blood cells: (a) membrane morphology; (b) nanoparticle morphology; Figure S4. Complete blood count (CBC) parameters of mice after 12-week subcutaneous implantation of PHA membranes; Figure S5. Serum biochemical parameters indicating liver and kidney function after 12-week implantation.; Figure S6. Representative H&E staining of underlying skeletal muscle tissue at 12 weeks. Upper panel: low magnification overview (scale bar = 500 μm); lower panel: high magnification detail of boxed regions (scale bar = 50 μm). (a) PBS control, (b) PHB, (c) P34HB 5%; Figure S7. Representative H&E staining of underlying skeletal muscle tissue at 12 weeks. Upper panel: low magnification overview (scale bar = 500 μm); lower panel: high magnification detail of boxed regions (scale bar = 50 μm). (d) P34HB 15%, (e) PHBV 5%, and (f) PHBV 15%; Figure S8. Complete blood count parameters of mice after intramuscular injection of PHA nanoparticles; Figure S9. Serum biochemical parameters after intramuscular injection of PHA nanoparticles. Table S1. Assignment of characteristic FTIR absorption bands for PHB and its copolymers.

## Abbreviations

The following abbreviations are used in this manuscript:

3HB	3-hydroxybutyrate
3HV	3-hydroxyvalerate
4HB	4-hydroxybutyrate
ALT	Alanine aminotransferase
ALP	Alkaline phosphatase
ANOVA	Analysis of variance
AST	Aspartate aminotransferase
CCK-8	Cell Counting Kit-8
Cr	Creatinine
DSC	Differential scanning calorimetry
DMEM	Dulbecco’s Modified Eagle Medium
DLS	Dynamic light scattering
EDTA-K_2_	Ethylenediaminetetraacetic acid dipotassium salt
FBS	Fetal bovine serum
FDA	Food and Drug Administration
FTIR	Fourier transform infrared
HCT	Hematocrit
H&E	Hematoxylin and eosin
HGB	Hemoglobin
HEPES	4-(2-hydroxyethyl)-1-piperazineethanesulfonic acid
HFF	Human foreskin fibroblast
HUVEC	Human umbilical vein endothelial cell
MCH	Mean corpuscular hemoglobin
NEAA	Non-essential amino acids
OD	Optical density
P/S	Penicillin/streptomycin
PBS	Phosphate-buffered saline
PLT	Platelet
PHB	Poly(3-hydroxybutyrate)
PHBV	Poly(3-hydroxybutyrate–co–3-hydroxyvalerate)
P34HB	Poly(3-hydroxybutyrate–co–4-hydroxybutyrate)
PHBHHx	Poly(3-hydroxybutyrate–co–3-hydroxyhexanoate)
P4HB	Poly(4-hydroxybutyrate)
PCL	Polycaprolactone
PHA	Polyhydroxyalkanoate
PLA	Polylactic acid
PLGA	Poly(lactide–co–glycolide)
PDI	Polydispersity index
PVA	Poly(vinyl alcohol)
RBC	Red blood cell
SEM	Scanning electron microscopy
UV	Ultraviolet
WBC	White blood cell
