# Supplementary material for: Systematic Evaluation of Structure–Property–Biocompatibility Relationships of Polyhydroxyalkanoate Copolymers for Advanced Veterinary Applications
Source: Molecules. 2026 Jul 6;31(13):2375. doi: 10.3390/molecules31132375 (PMC13363577; doi:10.3390/molecules31132375)
Supplement: Supplementary file 1 [file molecules-31-02375-s001.zip › molecules-4371114-supplementary.pdf]

Supplementary Materials

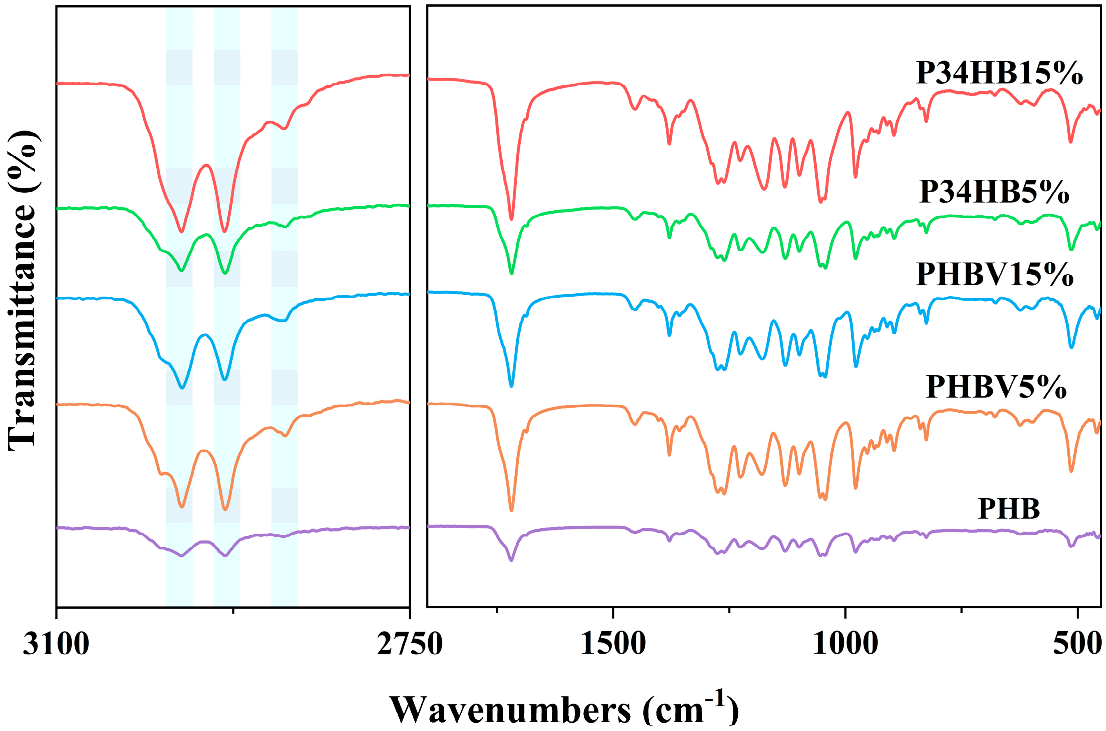

Figure S1. FTIR spectra of PHB and its copolymers in the range of 3100–500 cm^{-1}.

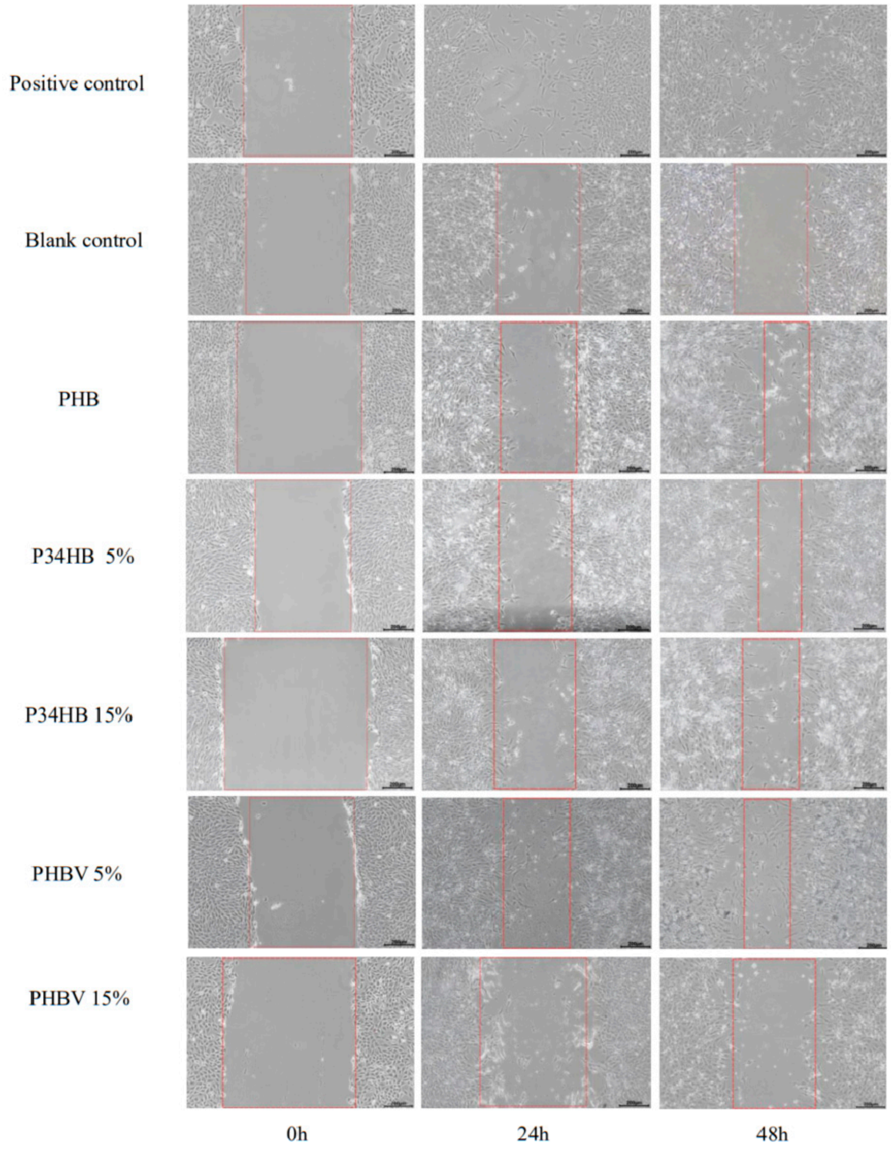

Figure S2. Representative images of HUVEC scratch wound healing at 0, 24, and 48 h. Scale bar = 200 μm. Poly(3-hydroxybutyrate) (PHB); poly(3-hydroxybutyrate-co-4-hydroxybutyrate) containing 5 mol% 4-hydroxybutyrate (P34HB 5%); poly(3-hydroxybutyrate-co-4-hydroxybutyrate) containing 15 mol% 4-hydroxybutyrate (P34HB 15%); poly(3-hydroxybutyrate-co-3-hydroxyvalerate) containing 5 mol% 3-hydroxyvalerate (PHBV 5%); and poly(3-hydroxybutyrate-co-3-hydroxyvalerate) containing 15 mol% 3-hydroxyvalerate (PHBV 15%).

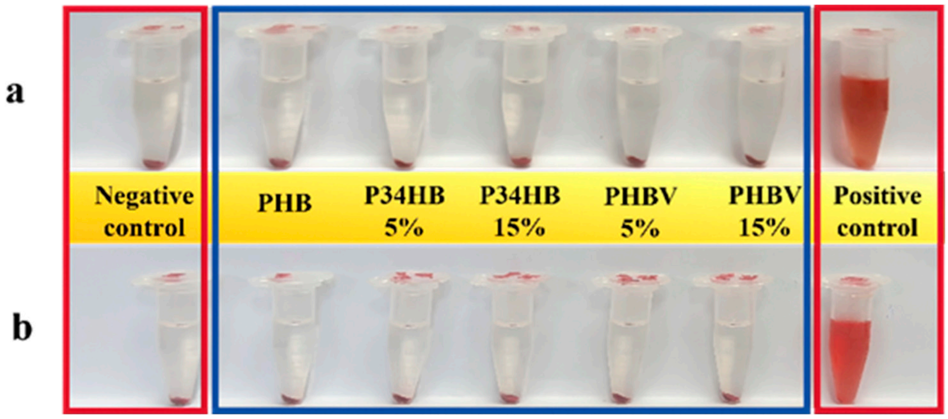

Figure S3. Nanoparticle morphology after incubation. Representative images of hemolysis assay after incubation with red blood cells: (**a**) membrane morphology; (**b**) nanoparticle morphology.

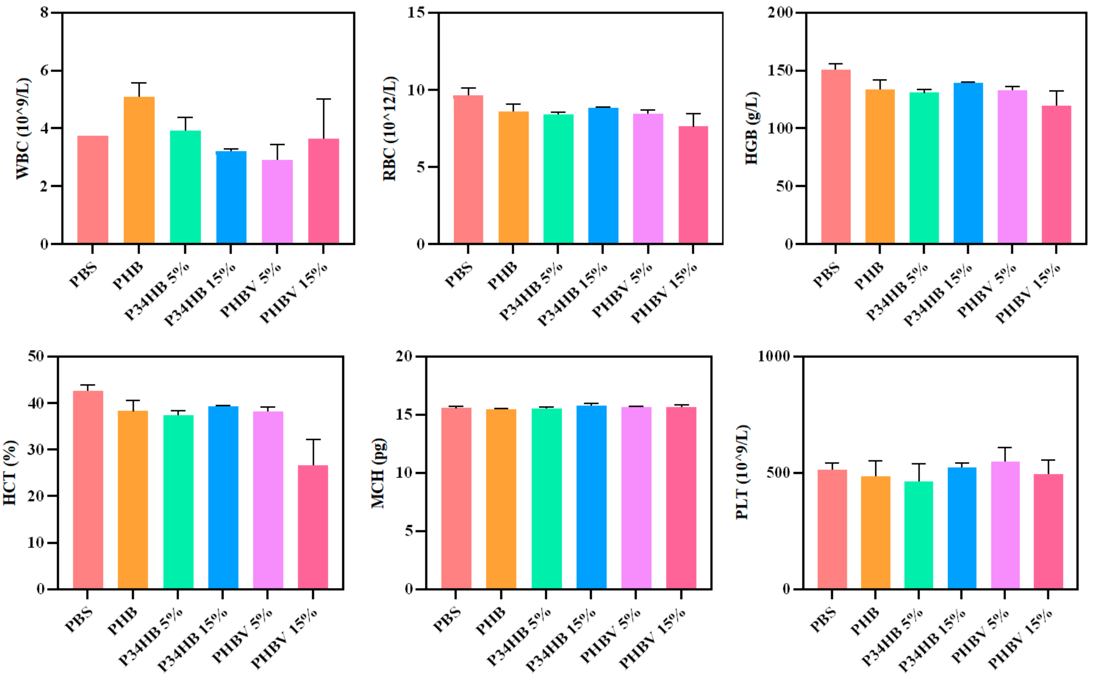

Figure S4. Complete blood count (CBC) parameters of mice after 12-week subcutaneous implantation of PHA membranes.

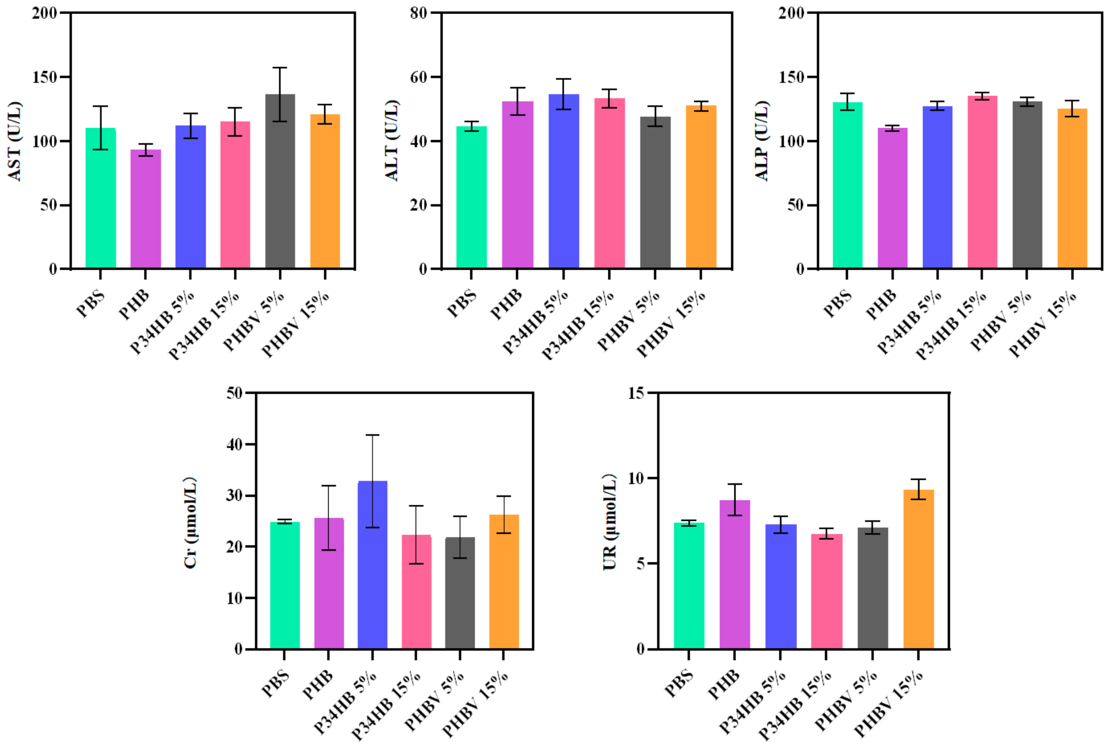

Figure S5. Serum biochemical parameters indicating liver and kidney function after 12-week implantation.

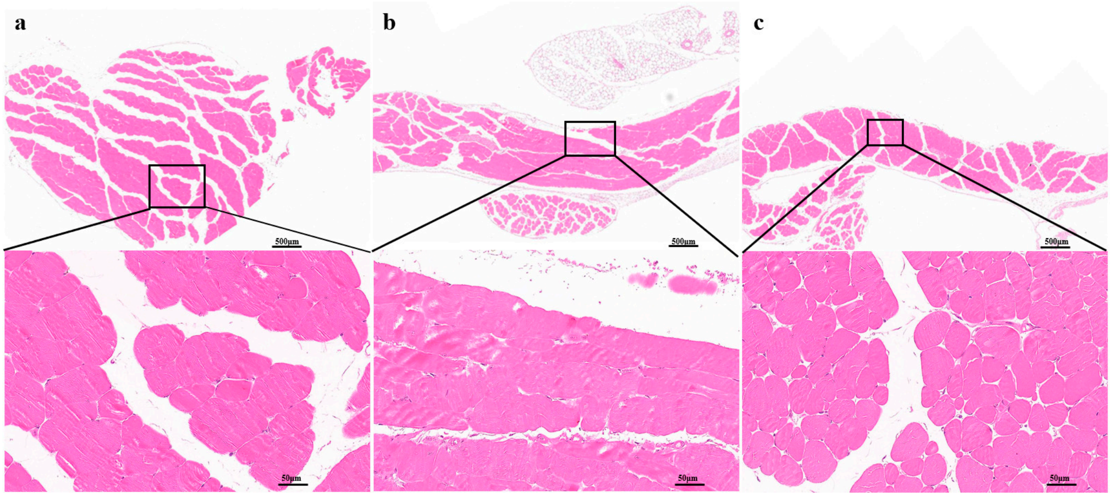

Figure S6. Representative H&E staining of underlying skeletal muscle tissue at 12 weeks. Upper panel: low magnification overview (scale bar = 500 μm); lower panel: high magnification detail of boxed regions (scale bar = 50 μm). (**a**) PBS control, (**b**) PHB, (**c**) P34HB 5%.

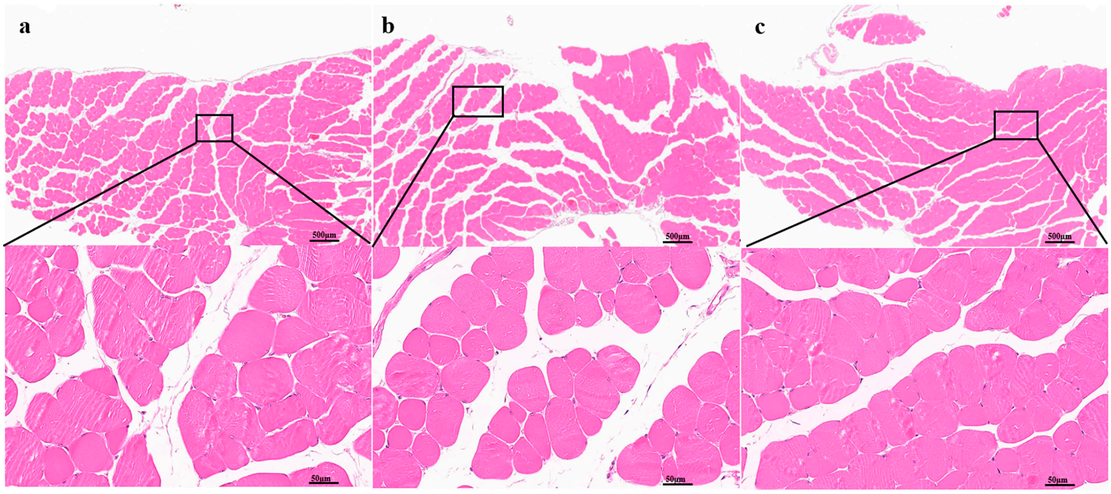

Figure S7. Representative H&E staining of underlying skeletal muscle tissue at 12 weeks. Upper panel: low magnification overview (scale bar = 500 μm); lower panel: high magnification detail of boxed regions (scale bar = 50 μm). (**a**) P34HB 15%, (**b**) PHBV 5%, and (**c**) PHBV 15%.

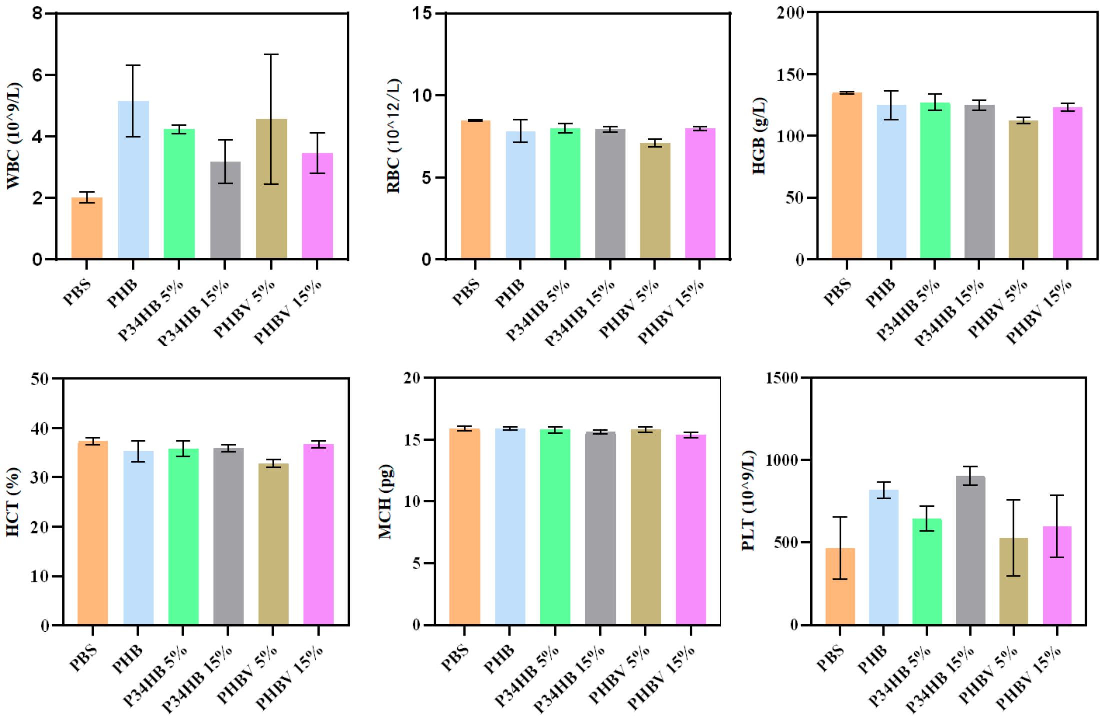

Figure S8. Complete blood count parameters of mice after intramuscular injection of PHA nanoparticles.

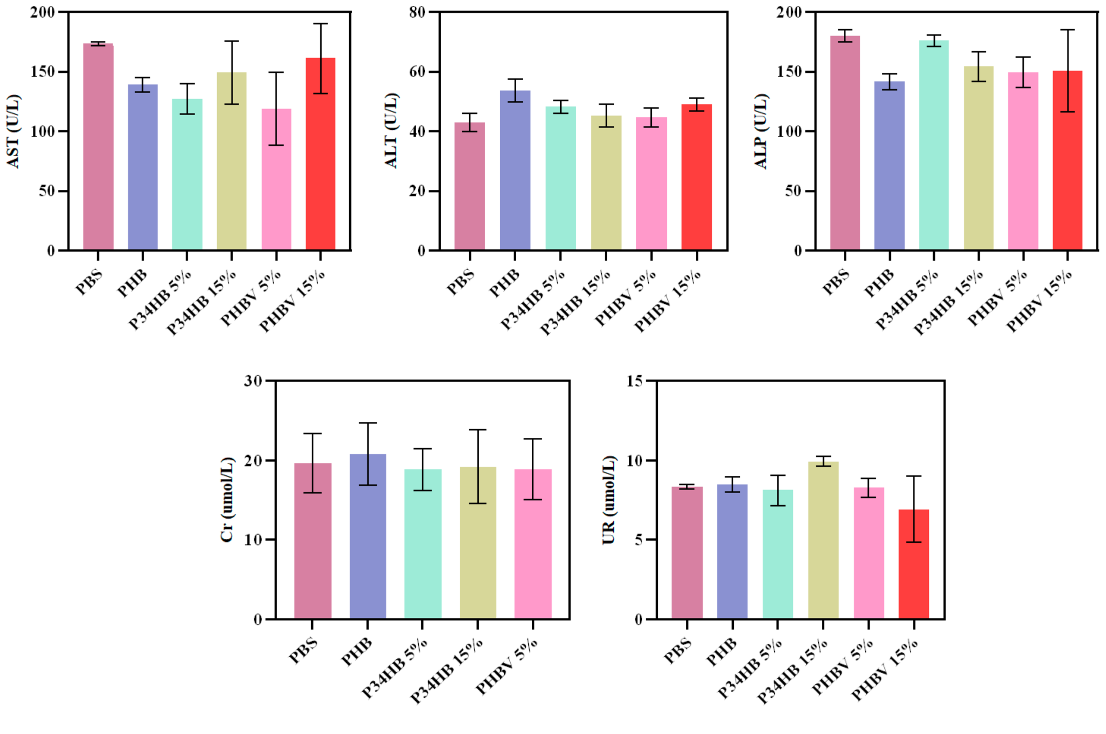

Figure S9. Serum biochemical parameters after intramuscular injection of PHA nanoparticles.

Table S1. Assignment of characteristic FTIR absorption bands for PHB and its copolymers.

Sample	CH2 and CH3 (cm^{-1})	C=O (cm^{-1})	C–O–C (cm^{-1})
PHB	2976，2932	1718.746	1275，1178，1099
PHBV 5%	2975，2932，2873	1718.746	1274，1179，1099
PHBV 15%	2975，2932，2873	1718.746	1274，1178，1099
P34HB 5%	2976，2933，2874	1718.264	1274，1178，1099
P34HB 15%	2976，2933，2874	1718.746	1273，1174，1099
